# Long-term effects of neuromuscular exercise therapy and the need for surgical conversion in wrist osteoarthritis: 24-month results from a randomized controlled trial

**DOI:** 10.1186/s12891-025-09463-5

**Published:** 2025-12-30

**Authors:** Sara L. Larsson, Elisabeth Ekstrand, Lars B. Dahlin, Anders Björkman, Elisabeth Brogren

**Affiliations:** 1 Department of Hand Surgery, Skåne University Hospital, Malmö, Sweden; 2 Department of Translational Medicine - Hand Surgery, Lund University, Malmö, Sweden; 3 Department of Biomedical and Clinical Sciences, Linköping University, Linköping, Sweden; 4 Department of Hand Surgery, Institute of Clinical Sciences, Sahlgrenska University Hospital, Sahlgrenska Academy, University of Gothenburg, Gothenburg, Sweden; 5 Department of Health Sciences, Lund University, Lund, Sweden; 6 Department of Translational Medicine, Lund university – Hand Surgery, Skåne University Hospital, Jan Waldenströms gata 5, Malmö, SE-205 03 Sweden

**Keywords:** Wrist osteoarthritis, Exercise therapy, Patient education, Self-management, Surgery conversion

## Abstract

**Background:**

Wrist osteoarthritis can cause persistent pain, reduced joint mobility, and difficulty performing daily activities, negatively impacting quality of life. Although patient education and exercise therapy are recommended as first-line treatments for osteoarthritis in other joints, evidence supporting these strategies for wrist osteoarthritis remains limited. This randomized controlled trial aimed to evaluate the long-term effects of neuromuscular exercise therapy, designed to improve joint strength and movement control, compared with range-of-motion exercises in individuals with wrist osteoarthritis, including the need for surgical conversion over 24 months.

**Methods:**

Forty-eight participants with symptomatic wrist osteoarthritis were randomly assigned to a 12-week self-management program. Participants received either a neuromuscular exercise therapy program (intervention group) or a program focused solely on range-of-motion exercises (control group). The primary outcome was the Patient-Rated Wrist Evaluation (PRWE) questionnaire, and secondary outcomes included grip strength, the Disabilities of the Arm, Shoulder, and Hand (DASH) questionnaire, pain ratings, and overall patient-rated improvement. Outcomes were assessed at baseline, 6 months, and 12 months, and surgical conversion was recorded from medical records at 24 months.

**Results:**

No statistically significant between-group differences were observed for the PRWE questionnaire or any secondary outcomes at 6 or 12 months. The calculated effect sizes for PRWE were 0.018 at 6 months and 0.0073 at 12 months, indicating very small effects and supporting the lack of clinically meaningful differences between the groups. Similarly, surgical conversion rates at 24 months did not differ significantly between the groups, although rates were relatively low in both the intervention group (21%) and the control group (17%).

**Conclusions:**

We found no clinically meaningful differences between the neuromuscular exercise therapy program and range-of-motion training in the treatment of wrist osteoarthritis at 6 and 12 months. Nevertheless, few participants in either group required surgery at 24 months, indicating that both exercise programs may contribute to symptom management over time. Further studies are warranted to refine and evaluate exercise-based interventions for the long-term management of wrist osteoarthritis.

**Trial registration:**

ClinicalTrials.gov, NCT05367817. Retrospectively registered on 27/04/2022. https://clinicaltrials.gov.

## Background

Individuals with wrist osteoarthritis (OA) often experience pain, joint-stiffness, and pain-associated psychological distress that reduce quality of life [1], most commonly resulting from traumatic or degenerative scapholunate ligament injury (SLAC) [2], or a non-united scaphoid fracture, termed Scaphoid Non-union Advanced Collapse (SNAC) [3]. Osteoarthritis management typically follows a patient-centred, stepwise approach, starting with education and exercise therapy, and progressing to surgery only if conservative treatment fails [4]. However, in contrast to OA of the knee, hip, and hand, wrist OA is relatively uncommon and has received limited attention concerning first-line management strategies, such as education and exercise therapy.

Wrist OA is a chronic condition without a cure, but patients may benefit from self-management strategies that help reduce symptoms and maintain quality of life [5]. Much of the existing literature on wrist OA has focused on surgical options [6], despite the fact that education and exercise therapy are recommended as first-line treatments for OA in most other joints. Furthermore, increasing surgical waiting times across healthcare systems have led to growing interest in prehabilitation to help preserve or enhance functional capacity prior to surgery and, in some cases, to delay or avoid the need for surgical intervention [7, 8]. Established rehabilitation programs exist for knee, hip, and hand OA, where patient education and exercise are offered as first-line treatments, and surgery is considered only if these conservative measures fail [9–11]. In contrast, a similar structured approach is lacking for individuals with painful wrist OA, where surgical interventions often are used as first-line treatment, with sometimes unsatisfactory results [12].

To investigate exercise therapy as a first-line intervention for wrist OA, we recently conducted a randomized controlled trial (RCT) in which 48 patients with symptomatic wrist OA participated, evaluating whether a self-managed neuromuscular exercise therapy program was superior to a range-of-motion training program [13]. Neuromuscular exercise therapy was chosen as the intervention because it targets functional re-learning and strengthening of the musculoskeletal system, with the aim of improving wrist stability and enabling pain-free use during daily activities [14, 15]. Since impairments in neuromuscular control, coordination, and stability have been suggested to be present in individuals with wrist OA [16], addressing these deficits was considered clinically relevant. Neuromuscular exercise programs are also widely used in the management of knee and hip OA, further supporting their relevance as an intervention in wrist OA [4, 17]. Range-of-motion exercises served as the control because they represent a basic and widely recommended starting point in conservative rehabilitation. Both groups were provided with the same core treatment package: patient education, orthosis use, and a 12-week home-based exercise program, including range-of-motion exercises, supported by scheduled follow-up assessments. The only difference between the groups was the inclusion of neuromuscular exercises in the intervention group, enabling us to isolate the specific therapeutic effect of this exercise approach on wrist OA. The programs were evaluated at 12 weeks, and no significant differences were observed between the groups in terms of pain reduction or functional improvement as reported in our previously published trial [18]. However, both exercise therapy and the application of self-management principles to support lasting lifestyle change are gradual strategies that require sustained effort before meaningful benefits become apparent [19]. Moreover, evaluating the potential of such first-line treatments to reduce the need for surgery necessitates sufficiently long follow-up periods. Therefore, this study aimed to assess the long-term effects of the previous RCT [18] by comparing outcomes between neuromuscular exercise therapy and range-of-motion exercises at 6 and 12 months, as well as the need for conversion to surgery at 24 months.

## Methods

### Study design, setting and population

This single-blinded superiority randomized controlled trial involved patients with symptomatic wrist OA treated at the Department of Hand Surgery, Skåne University Hospital in Malmö, Sweden, and was conducted and reported in accordance with the Consolidated Standards of Reporting Trials (CONSORT) guidelines [20]. Inclusion criteria were radiographically confirmed and symptomatic wrist OA—SLAC and SNAC stage 1–3 [2], and age ≥ 18 years. Exclusion criteria were patients with other diseases or disorders that could affect arm and hand function, wrist OA secondary to avascular necrosis of carpal bones, previous surgery to the wrist, intraarticular cortisone injection in the wrist within the last 3 months, and language or cognitive difficulties that could hinder the participant’s ability to complete the questionnaires. Eligible patients were randomly assigned in a 1:1 ratio to either exercise therapy with neuromuscular exercises (intervention) or range-of-motion exercises (control) using block randomization (block size = 10) generated by an independent occupational therapist. The personnel responsible for enrolling and assigning participants had no access to the allocation sequence, which was concealed in sequentially numbered, opaque, sealed envelopes opened by the treating physiotherapist at baseline. The Declaration of Helsinki was followed, and the study was approved by the Swedish Ethical Review Authority, Dnr 2019–02437 and retrospectively registered at ClinicalTrials.gov on 10/05/2022 (identification number NCT05367817). No important changes to the trial design, outcomes, or analyses were made after the trial commenced. All outcomes and analyses were prespecified in the trial protocol [13].

### Patient and public involvement

Patients or members of the public were not involved in the design, conduct, analysis, or reporting of this randomized controlled trial.

### Blinding

Participants took part in two different training programs and were not explicitly informed of their group allocation; however, it is possible that some participants may have inferred their assignment based on the type of exercises performed and were therefore not fully blinded to group allocation. Nevertheless, the groups were kept similar in format regarding patient education, orthosis use, and scheduled follow-ups to minimize potential bias and help maintain blinding. All evaluations at baseline and the 6- and 12-month follow-ups were conducted by an experienced physiotherapist who was blinded to group allocation. The blinded assessor performed all evaluations without access to participants’ medical records to avoid potential bias in outcome assessment.

### Treatment interventions

Both groups received verbal and written information on wrist OA pathophysiology, the rationale for exercise therapy, self-management strategies, and principles for activity modification. In addition, they were provided with a stable wrist orthosis to wear during pain-provoking activities and, if needed, at night. The two treatment programs were designed to be self-managed by the participants, with regular check-ups at the clinic. Participants received instructions for their assigned exercise program—neuromuscular exercise therapy (intervention) or range-of-motion exercises (control)—on the same day as their baseline assessment. The structured home-based exercise programs were carried out twice daily for 12 weeks and involved performing exercises with controlled movements. Adherence was emphasised, and participants were followed up by the treating physiotherapist at 2, 6, and 12 weeks in the clinic, with additional phone follow-ups at 4 and 8 weeks.

#### Intervention group

Received structured education, a wrist orthosis, range-of-motion exercises, and neuromuscular exercises targeting coordination, wrist stability, and strength, aiming to restore functional, pain-free use.

#### Control group

Received structured education, a wrist orthosis, and range-of-motion exercises only.

A detailed description of the structured patient education, the self-management strategies, and the exercises included in both programs has previously been published in a study protocol [13].

Exercise therapy is a non-invasive treatment method with no expected severe adverse events. However, a slight increase in symptoms can occasionally occur during exercise. This was monitored at the clinical follow-ups, and individual adjustments were made when necessary.

### Outcome measures

**Fig. 1:**
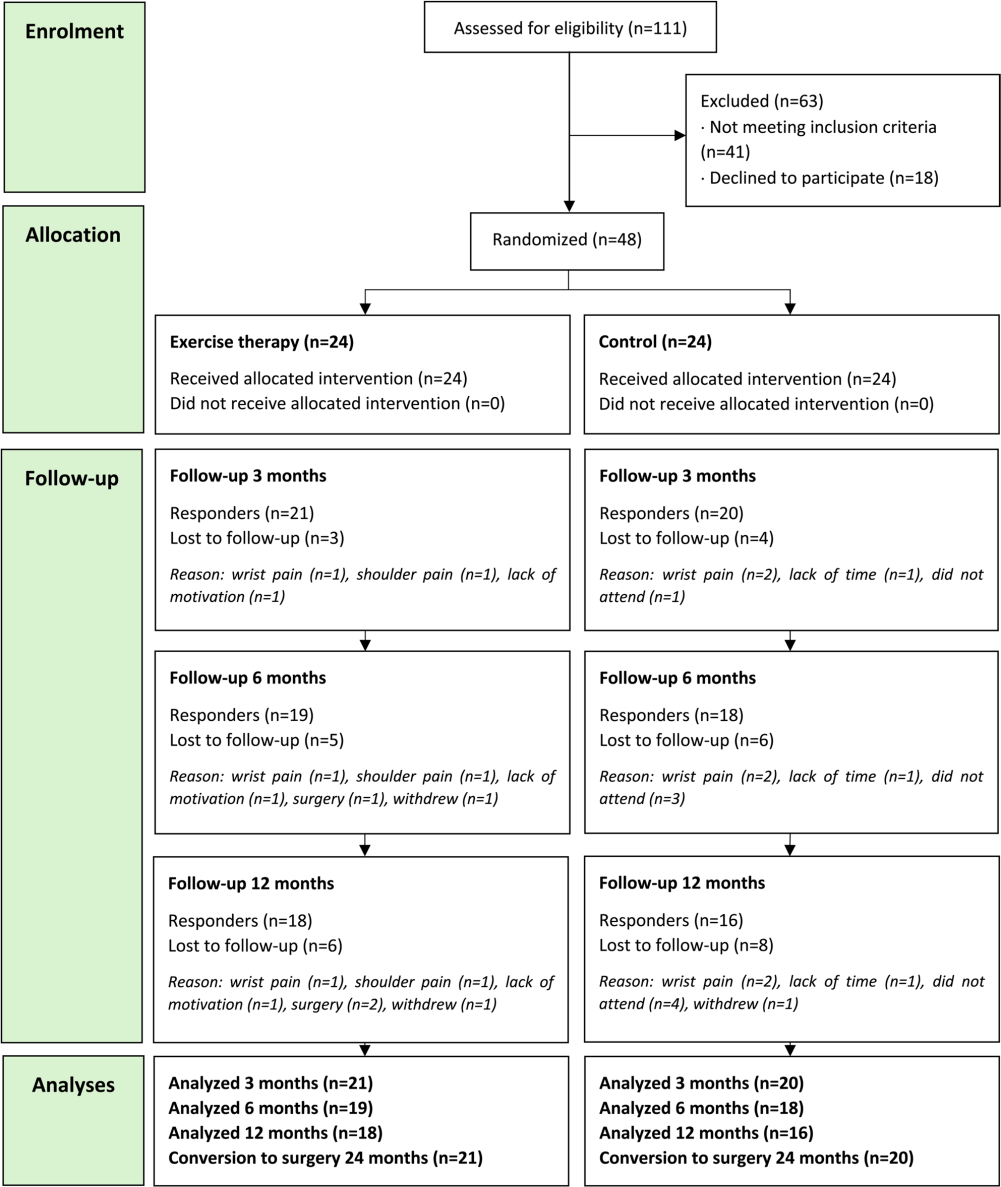
The Consolidated Standards of Reporting Trials (CONSORT) flow diagram of trial enrolment and follow-ups

Participants were followed for 12 months with evaluations at baseline, 3, 6 and 12 months after inclusion (Fig. 1). Conversion to surgery was evaluated by reviewing medical records for the number of participants who underwent traditional salvage procedures for wrist OA or were on the waiting list for these surgical interventions 24 months after inclusion. The Patient-Rated Wrist Evaluation (PRWE) [21] was our primary outcome measure, assessing wrist-specific pain and function on a scale from 0 to 100, where 100 indicates the greatest level of disability. Secondary outcomes were grip strength [22], the Numerical Pain Rating Scale (NPRS) [23], the Disabilities of the Arm, Shoulder, and Hand (DASH) questionnaire [24], and Global Rating of Change (GROC) [25]. Three aspects of pain—at rest, on motion without load, and on load—were rated with NPRS, ranging from 0 (no pain) to 10 (worst pain imaginable). On the DASH score, 100 represents the most severe disability, and 0 means no disability. At the 12-month follow-up, participants rated their overall change on the GROC scale, ranging from − 5 (much worse) to + 5 (much better), with 0 indicating no change with the question: “Regarding your wrist problems, how would you describe your wrist now compared to before the exercise therapy treatment?” The patient-reported outcome measures (PROMs) used in this study are validated Swedish versions, with PRWE, DASH, and NPRS psychometrically evaluated specifically for wrist OA [26].

### Sample size and statistical analysis

The sample size calculation specified 48 participants to be included in the study. The calculation used a minimal clinically important difference of 12.5 for PRWE, derived from pervious relevant studies [27, 28], a standard deviation of 14 (estimated in consultation with a statistician), power of 0.8, significance level of 0.05, and a 20% dropout rate. All randomized participants were included in the analysis according to their assigned groups (intention-to-treat principle). Non-parametric tests were used for the analyses since the data were not normally distributed, as confirmed by the Shapiro-Wilk test. Group differences were analysed using the Mann–Whitney U test, while within-group differences were assessed with the Wilcoxon signed-rank test. For group comparisons of our primary outcome PRWE at 6 and 12 months, effect sizes (η^2^) were derived from the Mann-Whitney U statistics. The Chi-square test was used to compare baseline characteristics. 95% confidence intervals (CIs) for categorical variables were calculated using the exact (binomial) method to provide precise estimates of proportions. For non-parametric variables, bootstrap resampling (1,000 iterations with replacement) was estimated, based on the percentile method. No interim analyses were performed, as the trial was designed with a relatively small sample size, a short recruitment and follow-up period, and no formal stopping rules. Given that the intervention—exercise therapy—is non-invasive and considered low risk, interim analyses were not warranted and could have unnecessarily increased the risk of type I error inflation. Complete case analysis was deemed the most appropriate approach, as recommended by a statistician, due to the relatively small baseline sample size. Missing data were therefore handled using complete case analysis. Only participants with available data at each time point were included in the analyses, and the number of participants with missing data per group is reported (Fig. 1). Data were analysed using IBM SPSS Statistics version 29 (IBM Corporation, Armonk, NY, USA). Statistical significance was set at *p* < 0.05.

## Results

### Participant enrolment

Of 111 patients assessed for eligibility, 48 were included and randomized, with 24 allocated to each group (Fig. 1). Participant recruitment occurred between October 2019 and March 2023. Due to some dropouts and participants lost to follow-up, 41 participants were included in the 3-month analysis (reported in our previous study [18]) while 37 were analysed at 6 months and 34 at 12 months (Fig. 1). Conversion to surgery was analysed on the 41 participants who completed the 12-week treatment program 24 months after inclusion.

### Delivery of the treatment programs and safety

Both the intervention and control programs were delivered by the same physiotherapist and implemented as intended, although adherence was not formally assessed. Participants continued their usual pain medications, such as paracetamol or NSAIDs, as needed, while opioids and intra-articular corticosteroid injections in the wrist were prohibited. No harms or unintended events occurred in either group during the trial.

### Baseline characteristics

At baseline, the groups did not differ with respect to demographic characteristics, patient-reported wrist function, pain intensity, or grip strength (Table 1). The most frequently observed underlying cause of OA was SLAC wrist, and there were more men included in both groups. The majority in both groups had SLAC/SNAC grades 2-3. Baseline comparisons between participants who completed the 12-month follow-up (responders) and those lost to follow-up showed a significantly higher proportion of SNAC wrists and OA in the dominant wrist among participants lost to follow-up (Table 2). No other significant differences were found in baseline characteristics, including patient-reported wrist function, pain levels, or grip strength (Table 2).

**Table 1:** Baseline characteristics of participants with wrist osteoarthritis, comparing the intervention (exercise therapy) and control groups

Characteristics	Exercise therapy group (*n* = 24)	(95% CI)	Control group (*n* = 24)	(95% CI)	*p*-value
Age, median [IQR]	63 [55–69]	(59–67)	66 [56–70]	(62–68)	0.45
Sex, male, n (%)^∗^	20 (83)	(64–93)	20 (83)	(64–93)	0.65
Type of OA, SLAC, n (%)^∗^	19 (79)	(60–91)	23 (96)	(80–99)	0.08
OA grade, n (%)^∗^					0.30
Grade 1	0 (0)	NA	3 (12.5)	(4–31)	
Grade 2	10 (42)	(25–61)	9 (37.5)	(21–57)	
Grade 3	12 (50)	(31–69)	9 (37.5)	(21–57)	
Grade 4	2 (8)	(2–26)	3 (12.5)	(4–31)	
Affected wrist, dominant, n (%)^∗^	15 (63)		20 (83)		0.10
PRWE	51 [33–67]	(39–58)	56 [40–64]^**^	(44–62)	0.54
DASH	31 [21–41]	(23–39)	36 [28–50]^**^	(30–49)	0.29
NPRS					
- At rest	3 [1–5]	(2–5)	3 [1–5]^**^	(2–4)	0.86
- On motion	7 [4–8]	(5–8)	5 [3–8]^**^	(4–7)	0.37
- On load	8 [7–9]	(8–9)	8 [5–8]^**^	(5–8)	0.07
Grip strength†	28 [23–36]	(24–35)	26 [18–37]	(19–32)	0.70

Values are numbers (n), medians, interquartile ranges [IQR], percentages (%) and 95% Confidence Intervals (CI)

*OA* Osteoarthritis, *SLAC *Scapholunate Advanced Collapse, *PRWE* Patient-Rated Wrist Evaluation, *DASH* Disabilities of the Arm, Shoulder and Hand, *NPRS* Numerical Pain Rating Scale, *NA* Not Applicable

†Measured with Jamar on the affected hand, expressed in kilograms (kg)

^*^The 95% confidence intervals refer to the proportion (%) and not to the absolute numbers (n)

^**^One missing

**Table 2:** Comparison of baseline characteristics between responders (participants who completed follow-up) and those lost to follow-up at 12 months in participants with wrist osteoarthritis

Characteristics	Responders (*n* = 34)	(95% CI)	Lost to follow-up (*n* = 14)	(95% CI)	*p*-value
Age, median [IQR]	65 [59–69]	(62–67)	63 [49–69]	(52–68)	0.36
Sex, male, n (%)^∗^	28 (82)	(70–94)	12 (86)	(52–92)	0.78
Exercise therapy group, n (%)^∗^	18 (53)	(37–69)	6 (43)	(21–67)	0.53
Type of OA, SLAC, n (%)^∗^	32 (94)	(81–98)	10 (71)	(45–88)	**0.03**
OA grade, n (%)^∗^					0.81
Grade 1	1 (3)	(2–19)	2 (14)	(1–32)	
Grade 2	15 (44)	(24–55)	4 (29)	(21–67)	
Grade 3	14 (41)	(29–61)	7 (50)	(21–67)	
Grade 4	4 (12)	(5–27)	1 (7)	(1–32)	
Affected wrist, dominant, n (%)^∗^	22 (65)	(37–69)	13 (93)	(69–99)	**0.048**
PRWE	53 [39–69]	(47–62)	52 [34–57]	(35–56)	0.43
DASH	35 [26–50]	(31–40)	30 [24–45]	(25–42)	0.47
NPRS					
- At rest	3 [1–5]	(2–5)	2 [1–4]	(2–3)	0.34
- On motion	5 [4–8]	(5–7)	7 [4–8]	(5–8)	0.40
- On load	8 [6–8]	(7–8)	8 [6–9]	(7–9)	0.43
Grip strength†	28 [19–35]	(24–31)	26 [20–40]	(22–38)	0.50

Values shown in bold are statistically significant (*p* < 0.05). Values are numbers (n), medians, interquartile ranges [IQR], percentages (%) and 95% Confidence Intervals (CI)

*OA* Osteoarthritis, *SLAC* Scapholunate Advanced Collapse, *PRWE* Patient Rated Wrist Evaluation, *DASH* Disabilities of the Arm, Shoulder and Hand, *NPRS* Numerical Pain Rating Scale

†Measured with Jamar on the affected hand, expressed in kilograms (kg)

^*^The 95% confidence intervals refer to the proportion (%) and not to the absolute numbers (n)

### Outcome measures

**Fig. 2:**
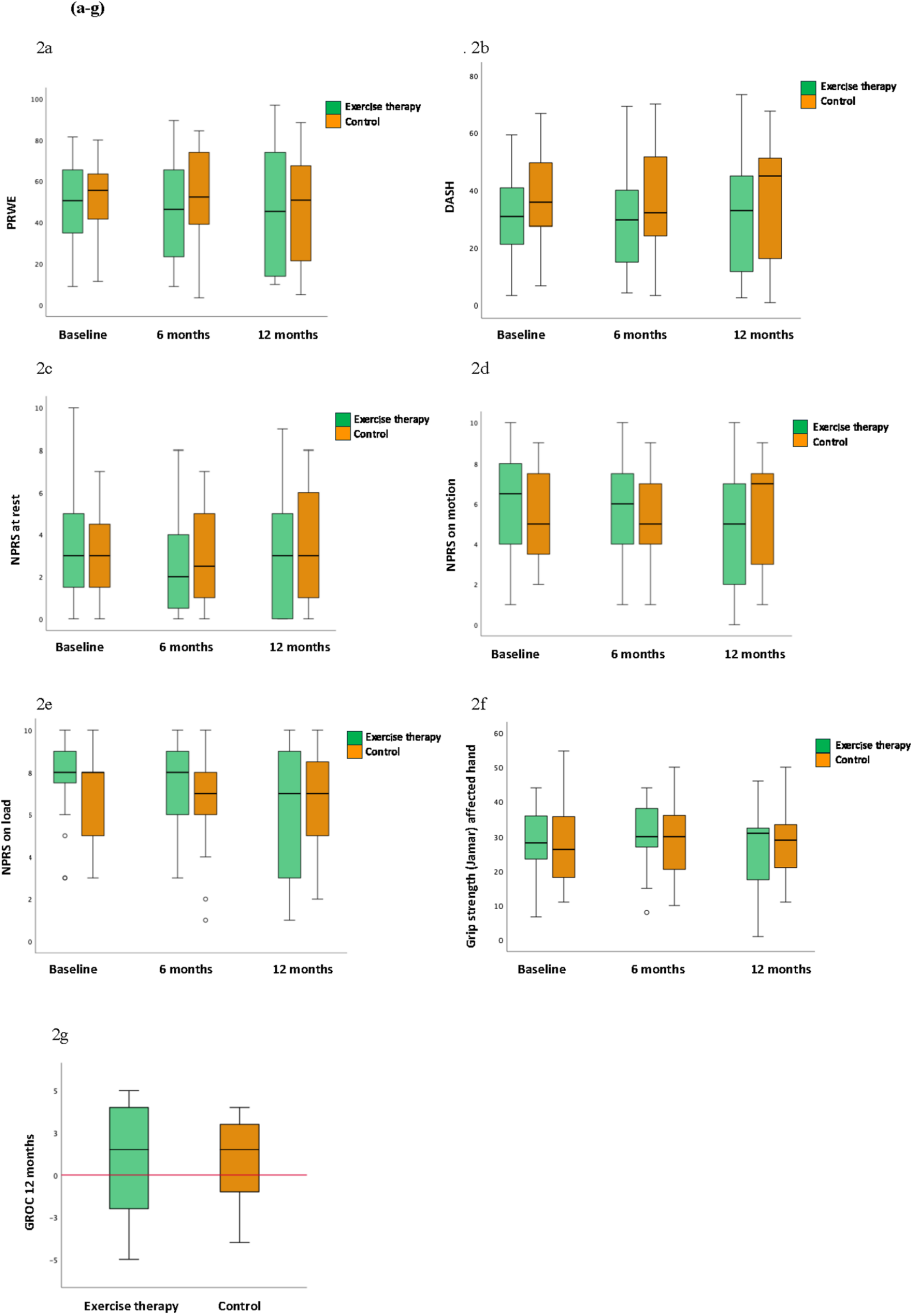
**a**-**g** Box plots showing medians, interquartile ranges [IQRs], and outlier values for the PRWE (Fig. 2a), DASH (Fig. 2b), NPRS at rest (Fig. 2c), NPRS on motion (Fig. 2 d), NPRS on load (Fig. 2e), grip strength affected hand expressed in kilograms (Fig. 2f) for the exercise therapy group (intervention) and the control group at baseline, 6-month, and 12-month follow-ups. The box plots in Fig. 2 g show the GROC scale values at the 12-month follow-up, with the red line representing no change. Boxes extend from the 25^th^ to the 75^th^ percentile of each group´s distribution of values. The whiskers denote the most extreme values within 1.5 interquartile range of the 25^th^ and 75^th^ percentile. Data points outside the whiskers’ boundary are plotted as outliers

Regarding our primary outcome measure, PRWE, we observed no statistically significant difference between the groups at 6 and 12 months. The calculated effect sizes were 0.018 at 6 months and 0.0073 at 12 months, indicating very small effects and supporting the lack of clinically meaningful differences between the groups. Additionally, there were no significant changes within each group from baseline to either 6 or 12 months (Fig. 2a). For our secondary outcomes, we found no statistically significant differences in patient-reported pain and function (NPRS and DASH) or grip strength between the exercise therapy group and the control group, nor any within-group differences from baseline to 6 and 12 months (Fig. 2b-f). Both groups showed a slight improvement in the GROC score after 12 months, but there was no significant difference between them (Fig. 2g). The results are presented in box plots (Fig. 2a-g).

### Conversion to surgery

No statistically significant between-group differences in conversion to surgery were observed 24 months after inclusion (21% in the neuromuscular exercise therapy group and 17% in the control group) (Table 3).

**Table 3:** Comparison of conversion to surgery between the exercise therapy group (intervention) and the control group in participants with wrist osteoarthritis 24 months after inclusion

	Exercise therapy group (*n* = 21)	Control group (*n* = 20)	*p*-value
Surgery, n (%)	5 (21)	4 (17)	0.82
Type of surgery, n (%)			0.73
- TWF	3 (13)	2 (8)	
- PRC	1 (4)	1 (4)	
- SPA	1 (4)	0 (0)	
- Denervation	0 (0)	1 (4)	

Conversion to surgery was defined as performed or planned surgery. Values are numbers (n) and percentages (%)

*TWF* Total Wrist Fusion, *PRC* Proximal Row Carpectomy, *SPA* Scaphoid Pseudarthrosis (screw fixation)

## Discussion

This is the first RCT to investigate exercise therapy specifically for wrist OA, in contrast to over 100 trials for knee OA [29], 18 RCTs for hip OA [30], and 14 RCTs for hand OA [31, 32]. In this extended follow-up of our previously published RCT [18] we found no significant differences between neuromuscular exercise therapy and range-of-motion training at 6 or 12 months. Thus, there was no long-term advantage of neuromuscular exercises over range-of-motion exercises. Similarly, no significant between-group differences were observed in surgical conversion rates at 24 months.

Although neuromuscular exercise therapy did not show clinically meaningful advantages over range-of-motion exercises, a potential beneficial effect cannot be excluded due to the small sample size. Exercise therapy may therefore still be considered a possible first-line management option for individuals with wrist OA, particularly as this is the first study to evaluate this approach. The evidence of exercise therapy is strongest for knee OA and moderate for hip OA, whereas the benefits for hand OA are more inconclusive. Exercise therapy is still widely recommended as a core treatment because of its potential to improve pain, function, and overall health [33], despite growing uncertainty regarding its clinical effectiveness in OA [34]. Importantly, recent guidelines emphasise the need for more research on non-weight-bearing joints, such as the wrist [33]—a gap our study has addressed.

The wrist, however, is biomechanically and structurally distinct from the knee and hip joints [35]. It consists of multiple small joints and lack large surrounding muscles for direct stabilisation, making it challenging to treat and assess. The neuromuscular exercise therapy program in this study was developed based on theoretical [15, 36] and clinical research [37–42] related to the rehabilitation of various wrist injuries. Similar to the design of our exercise therapy program with neuromuscular exercises, these studies focus on exercises targeting the affected wrist rather than the whole-body, progressively loaded, bilateral, and cardiovascular-engaging programs used in lower-limb OA [29, 30]. Consequently, the lack of effect observed in our trial may be attributed to the intervention’s narrow focus on the affected wrist and highlights the need for more comprehensive and progressive protocols.

In this study, most participants had SLAC/SNAC grade 2 or 3 and were treated in a tertiary care setting. Unlike patients with knee, hip, or hand OA—who typically receive early exercise therapy and joint protection strategies in primary care [43]—patients with wrist OA are often managed initially with pain medication and orthoses. If these measures fail, they are referred to specialised tertiary clinics. As a result, some participants in our trial may have had advanced OA stages less responsive to exercise therapy. Introducing exercise therapy earlier in the care pathway, including as a preventive strategy for post-traumatic wrist pain, may be more effective, similar to approaches used in managing traumatic knee injuries [44].

Although the neuromuscular exercise therapy program showed limited effects, the conversion rate to salvage wrist surgery was relatively low in both groups at 24 months. This contrasts with higher rates reported following partial wrist denervation—28% at 12 months by Swärdh et al. [45], and 24% at 18 months by Kadhum et al. [46]—suggesting that exercise therapy may support effective long-term symptom management and delay the need for surgical intervention. However, to reliably determine which first-line treatment—exercise therapy or partial wrist denervation—yields better outcomes and lower rates of conversion to surgery, a randomized controlled trial is warranted.

This is a novel approach to evaluate exercise therapy as a treatment for wrist OA, and as such, the study has certain limitations. The small sample size due to dropouts and participants lost to follow-up, increases the risk of type II errors. Missing data were handled using complete case analysis, which may have introduced selection bias if the missingness was not completely at random and related to unobserved outcomes. Additionally, our ability to assess the relationship between adherence and outcomes was limited by the absence of relevant data; variations in patients’ follow-through may have influenced the results. The observed effect sizes in our primary outcome (PRWE) were small and not clinically meaningful, and baseline differences in SNAC prevalence and osteoarthritis of the dominant wrist between responders and those lost to follow-up at 12 months may have introduced attrition bias, potentially affecting group comparability and the validity of the findings. The study design aimed to minimize performance bias by ensuring that both groups received equivalent levels of attention This included identical patient education, the same orthosis, and scheduled follow-ups by the same physiotherapist. However, this approach also reduced the intervention contrast between the two exercise programs. As a result, the similarity of the programs may have diluted any observable treatment effects, limiting our ability to detect true differences between the neuromuscular and range-of-motion interventions.

Nonetheless, to our knowledge, a key strength of this study is that it is the first to evaluate exercise therapy as a first-line treatment for wrist OA using a randomized controlled design with long-term follow-ups. Future research should focus on multi-centre studies to increase sample size and improve generalisability and should also target individuals with post-traumatic wrist injuries or early-stage wrist OA to help identify subgroups that that may respond more favorably to specific interventions. Neuromuscular exercise therapy may have greater potential earlier in the disease process, before structural changes become too advanced. Future research could also explore whether involvement of the dominant hand influences adherence or outcomes, as participants with OA in the dominant wrist may experience greater pain or functional limitations, potentially affecting engagement in exercise programs and follow-up. Furthermore, to evaluate the true effects of an exercise therapy program for wrist OA, future RCTs should include a control group that is not prescribed exercise as treatment or compare an exercise program with alternative pharmacological or surgical treatment options. In addition, since joint protection principles emphasize distributing load across several joints and using the strongest and most proximal joints for functional tasks [47], future exercise programs may benefit from including the entire upper extremity rather than focusing solely on the wrist. More individualized and goal-oriented approaches may also be needed, as symptom presentation and functional demands vary widely between patients. Finally, co-developing exercise programs together with patients could enhance feasibility, adherence, and relevance for everyday activities and should be explored in future research [48].

## Conclusions

No clinically meaningful differences were observed between the neuromuscular exercise therapy program and range-of-motion training in the treatment of wrist osteoarthritis at 6 and 12 months. However, few participants in either group required surgery at 24 months, suggesting that both programs may help manage symptoms over time. Further research is warranted to refine, optimize, and explore exercise-based interventions as non-invasive treatment strategies for wrist osteoarthritis. 

## Data Availability

The datasets generated and/or analyzed during the current study are not publicly available due to restrictions under the Swedish Public Access to Information and Secrecy Act ([https://www.government.se/information-material/2009/09/public-access-to-information-and-secrecy-act/] (https://www.government.se/information-material/2009/09/public-access-to-information-and-secrecy-act/)). Data may, however, be made available from the corresponding author upon reasonable request. Access requires special review and approval of the research project by both a national Ethics Committee and governmental data safety committees.

## Abbreviations

CONSORT: Consolidated Standards of Reporting Trials
DASH: Disabilities of the Arm, Shoulder, and Hand
GROC: Global Rating of Change
NPRS: Numeric Pain Rating Scale
NSAID: Non-steroidal Anti-inflammatory Drug
OA: Osteoarthritis
PRWE: Patient-Rated Wrist Evaluation
RCT: Randomized Controlled Trial
SLAC: Scaphoid Lunate Advanced Collapse
SNAC: Scaphoid Non-Union Advanced Collapse
